# Animal Welfare Assessment of Fattening Pigs: A Case Study on Sample Validity

**DOI:** 10.3390/ani10030389

**Published:** 2020-02-27

**Authors:** Mareike Pfeifer, Armin Otto Schmitt, Engel Friederike Hessel

**Affiliations:** 1 Department of Animal Sciences, Georg-August-University of Göttingen, Gutenbergstraße 33, D-37075 Göttingen, Germany; 2 Breeding Informatics, Georg-August-University of Göttingen, Margarethe von Wrangell-Weg 7, D-37075 Göttingen, Germany; 3 Center for Integrated Breeding Research (CiBreed), Georg-August-University of Göttingen, Albrecht-Thaer-Weg 3, D-37075 Göttingen, Germany; 4 Thünen-Institute of Agricultural Technology, Federal Research Institute for Rural Areas, Forestry and Fisheries, Bundesallee 47, D-38116 Braunschweig, Germany; engel.hessel@thuenen.de

**Keywords:** animal welfare evaluation, animal welfare indicators, random sampling, validity, feasibility

## Abstract

**Simple Summary:**

The welfare of farm animals is discussed in society and politics. In Germany, the Association for Technology and Structures in Agriculture developed a new guideline for the animal welfare assessment of fattening pigs. It is called *ANIMAL WELFARE INDICATORS: PRACTICAL GUIDE – PIGS* and contains 13 characteristics by which the welfare of an animal is assessed, so-called indicators, as well as instructions on how to collect those indicators. For reasons of feasibility, six of the indicators should not be collected for all fattening pigs in a herd, but for a sample. The question arises whether then the herd’s level of animal welfare is assessed with sufficient precision. For this reason, this study examines five strategies for collecting samples of the fattening pigs in a herd. The aim is to identify a feasible strategy that collects samples of high validity. However, the study shows that the result of the animal welfare assessment based upon samples can partly deviate considerably from the result of the assessment of the entire herd. Further studies are needed to identify the most feasible and valid method for collecting samples of pigs from a herd.

**Abstract:**

A guide for animal welfare assessment of fattening pigs recommends recording some of the indicators for a sample of the animals from a herd. However, it is not certain whether the herd’s level of welfare can be correctly judged using a random sample. Therefore, both the true prevalences of welfare indicators in a full census and the estimated prevalences of the indicators based upon simulated samples taken according to five strategies (termed S1 to S5) were determined. Deviations from the true level of animal welfare in the herd due to the sampling were recorded and analyzed. Depending on the strategy, between 12% and 43% of the samples over- or underestimated the true prevalences by more than 50%. The validity of the sampling strategies was evaluated using the normalized root-mean-squared error (NRMSE) and the relative bias (RB). In terms of accuracy, the strategies differed only slightly (between NRMSE = 0.13 for S2 and NRMSE = 0.19 for S4). However, the strategies varied more obviously regarding the bias (between RB = −0.0002 for S1 and RB = −0.0370 for S5). The described results are the outcome of an initial case study on the sample validity of the indicators and have to be verified using the data of more herds.

## 1. Introduction

The topic of animal welfare assessment is becoming more important because of the increased societal and political interest in farm animal welfare. In Germany, the Association for Technology and Structures in Agriculture [Kuratorium für Technik und Bauwesen in der Landwirtschaft e.V. (KTBL)] has made a proposal for assessing the welfare of sows, suckling pigs, weaning pigs, and fattening pigs in the publication *ANIMAL WELFARE INDICATORS: PRACTICAL GUIDE–PIGS* (available only in German) [1]. Comparable, internationally known approaches are the *Welfare Quality^®^ Animal Welfare Assessment Protocol for Growing Pigs* [2] and the *Bristol Welfare Assurance Programme* [3]. For the animal welfare assessment of fattening pigs, the KTBL guide contains a total of 13 indicators (twelve animal-based and one resource-based indicator) and instructions on how to collect them, i.e., the recommended frequency and moment of recording and evaluation for each indicator. The guide is primarily aimed at farmers but can also be used by veterinarians or advisors to assess the welfare of pigs on a voluntary basis. An essential precondition for an intensive use and acceptance of the guide by farmers, veterinarians, and advisors is a high degree of feasibility, which the authors of the guide have defined as a simple collection of the indicators with a minimum effort for survey and documentation and a positive cost-benefit ratio of conducting the animal welfare assessment in accordance with the guide [4]. To realize these conditions, the following procedure, among others, is recommended in the guide for the collection of several indicators: instead of a time- and cost-intensive recording for all the animals of a herd (full census), these indicators should be recorded for a sample of the animals [1]. This recommendation relates to the indicators tail lesions, faecal soiling, skin lesions, ear lesions, lameness, runts, and the presence of ectoparasites. Since none of the animals suffered from ectoparasites, this indicator was not included in this study. The other indicators contained in the guide were not taken into account in this study as well because they are intended to be recorded for the whole herd or for the husbandry system (resource-based indicators), so that sampling does not has to be applied. The six indicators used in the study are described in Table 1. Each of the indicators was assessed according to a two- or three-level rating scale. The KTBL recommendations for collecting these indicators are described in Section 2.2.

The question arises whether the recommendation to consider samples of the pigs from a herd for animal welfare assessment is admissible in terms of validity (sample validity). This means, how trustworthy and accurate is the level of animal welfare assessed by evaluating parts of the fattening pigs in a herd and how strong is the bias, i.e., the systematic under- or overestimation of the true animal welfare level of a herd because of the sampling, compared to a full census? In order to investigate this, a case study was done on the six indicators. For the sampling of the pigs, five different strategies were used and compared. The hypothesis was that the prevalence of the indicators after the evaluation of all the pigs in the herd (i.e., the true prevalence) corresponds to the prevalence of the indicators after the evaluation of the sampled pigs (i.e., the estimated prevalence) and therefore the use of sampling in welfare assessment is valid. In order to examine this hypothesis, the following three sub-questions were considered: (1) To what degree did the true and estimated prevalences of the KTBL indicators differ? (2) Which proportion of the simulated samples over- or underestimated the true prevalence of the KTBL indicators by 10%, 30%, or 50%? (3) Which of the five sampling strategies has the highest overall sample validity and feasibility?

## 2. Animals, Materials, and Methods

### 2.1. Determining The True Prevalences of the Animal Welfare Indicators

The welfare of the fattening pigs that were held at the Relliehausen Experimental Farm of the University Göttingen, Germany, was evaluated according to the six abovementioned KTBL indicators. The fattening pigs were conventionally kept in an insulated, forced-ventilated barn with a total of 800 fattening places. The barn was divided into four rooms of eight pens each. A pen had room for up to 25 fattening pigs. Each pen had a concrete slatted floor (slot width 17 mm, slat width 80 mm), an automatic liquid feeder (animal: feeding place ratio maximum 6.25:1), four drinking nipples, hay racks, and various different types of materials for environmental enrichment, for example, chains, wooden blocks, and jute bags. The floor space was at least 0.75 m^2^ per fattening pig. The rooms were run on an all-in/all-out basis. The routinely tail docked fatteners were taken from the farm’s own piglet production with an average stalling-in weight of 30 kg. The fattening pigs were sent to the slaughterhouse with a body weight of 118 kg, after an average fattening period of 87 days with an average daily weight gain of 900 g.

On the day of data collection, a total of 636 fattening pigs were kept on the farm. The distribution of the fattening pigs between the four rooms as well as their average live weights and the number of fattening days, which is the number of days since the pigs were moved to the fattening pens, are shown in Table 2. An assessor recorded the six KTBL indicators considered in this study for all pigs on the farm. The assessor was experienced in recording indicators regularly, but was not particularly accredited because no official training program for animal welfare assessment in accordance with the KTBL guide has yet been developed [5]. The assessment was done pen by pen. Once the assessor had entered a pen, each fattening pig was given a score for each of the six indicators. The indicators faecal soiling, skin lesions, and ear lesions were assessed for only one randomly chosen side of the body of a fattening pig according to the KTBL guide. For assessing the indicator lameness, the assessor made the pigs move a few steps.

### 2.2. Calculation of the Estimated Prevalences of the Animal Welfare Indicators

The data set containing the scores of the six KTBL indicators collected during the welfare evaluation of all 636 fattening pigs on the farm was transferred to R version 3.4 [6]. Thereafter, the scores of the indicators skin lesions and faecal soiling were transformed to the binary scoring structure as the other four KTBL indicators. For these two indicators, the initial scores zero and one were combined and classified as normal. In the following, fattening pigs that were rated with the highest score of an indicator, i.e., score two for the indicators skin lesions and faecal soiling and score one for the remaining four KTBL indicators were classified as problematic. Then, the data set was used to test the five strategies, which are described in Table 3. Strategy 5 (S5), which consists of the recommendations of the KTBL guide, is the reference strategy. In detail, the KTBL recommends the following [1]: In herds of up to 150 fattening pigs, the six indicators should be judged in all the animals. In herds with more than 150 fattening pigs, ten randomly chosen pig pens should be selected, taking into consideration the different age and weight classes but ignoring the isolation pens (for sick animals). The welfare of all the pigs in these pens should then be evaluated using all six indicators. However, in pens with more than 15 fattening pigs, the indicators skin lesions, ear lesions, and tail lesions should only be evaluated in 15 randomly chosen pigs per pen. If there are fewer than ten pig pens available, then the number of pigs evaluated per pen should be increased so that in the end a total of 150 fattening pigs have been evaluated with respect to these three indicators. Strategy 1 to 4 (S1 to S4) are different procedures for drawing samples from the fattening pigs and are regarded as alternatives to the reference strategy. For each strategy and indicator, n = 100,000 samples were drawn using the R-function ‘sample’. Pigs and pens, respectively, were drawn without replacement. For each sample, the selected number of pigs and the distribution of the scores of the indicators were determined and written in a separate file.

In order to ensure comparability, a similar number of fattening pigs was randomly chosen from the herd for each of the five strategies, i.e., the sizes of the samples were as identical as possible. The procedure for determining this sample size was as follows: Initially, 100,000 random samples were chosen according to the reference strategy S5. Within S5, the indicators were separated into two groups which were treated differently (Table 3). The indicators runts, faecal soiling, and lameness were collected for all fattening pigs in ten randomly chosen pens, whereas the indicators skin lesions, ear lesions and tail lesions were collected from 15 fattening pigs chosen randomly from these ten pens. On average, across all indicators, the 100,000 samples collected according to S5 resulted in a sample size of 167 fattening pigs. Consequently, this sample size was used for both S1 and S2. However, this size of the random samples for S3 and S4 could not be determined exactly due to the particular definition of these two strategies (see Table 3). With an average of 19.9 fattening pigs per pen (636 pigs, 32 pens), theoretically 8.4 pens would have had to be chosen for the collection of a sample size of 167 fattening pigs. For our simulations, a sample of 8 randomly chosen pens was used for both S3 and S4. This resulted in an average sample size of 159 fattening pigs for these two strategies.

For S2, S4, and S5, the different body weight classes should be taken into account proportionally during the sampling. As the fattening barn on the experimental farm is run on the all-in/all-out principle, this meant that each room only had fattening pigs in one age or weight class on the day of data collection. As a consequence, the following methods were used in order to fulfil the age/body weight class specification: In S2, the 167 fattening pigs were randomly chosen from all four rooms, with the number of pigs in the sample being proportional to the total number of pigs in the room (23 pigs from room 1, 46 pigs from both rooms 2 and 3, 52 pigs from room 4). For S4 and S5, two pens per room were randomly selected, and, for S5, two additional randomly selected pens from two different rooms were taken into account.

### 2.3. Statistical Analysis

A sampling strategy is valid and has a high quality when the results of the welfare evaluation based upon the random samples tallies with the animal welfare assessment for the whole herd, i.e., if there is as little difference as possible between the estimated and the true prevalences of the indicators. As a consequence, a descriptive comparison of the true and estimated prevalence of the six KTBL indicators was undertaken first. Additionally, the degree of deviation between the true and the estimated prevalence was calculated. To achieve this, the proportion of the 100,000 generated samples per strategy and indicator which over- or underestimated the true prevalence of the indicators by 10%, 30%, and 50% was determined. For example, if the true prevalence of an indicator is 5%, then all random samples that showed an estimated prevalence of less than 4.5% or more than 5.5% had under- or overestimated the true value by more than 10%. Both systematic and random deviations can be used to characterize differences between the true and the estimated prevalence of the indicators. Accordingly, the validity of the five sampling strategies was evaluated by the two measures of bias and accuracy defined as follows: the bias is a measure of systematic under- or overestimation of the true prevalence *p* of an indicator in a herd because of sampling. It is defined as the average difference between the sample estimates $\hat{p}$*_i_* of the prevalence and the true prevalence *p*. Here, *i* runs through all simulated samples 1 ≤ *i* ≤ 100,000 for a given indicator and a given sampling strategy. Thus,

(1) $$\mathrm{Bias}=\frac{1}{100\;000}\sum_{i=1}^{100\;000}\;\hat{p_{i}}-p$$

In order to account for the divergent values of the true prevalences for the indicators (from 1.1% for runts to 8.5% for ear lesions), the measure relative bias (RB), which is defined as the bias divided by the true prevalence, was chosen. Thus,

(2) $$\mathrm{RB}=\frac{\mathrm{Bias}}{\mathrm{True}\;\mathrm{prevalence}}$$

The root-mean-squared error (RMSE) is a measure of the average deviation between the estimates of the prevalence and the true prevalence. It is defined as follows:

(3) $$\mathrm{RMSE}=\sqrt{\frac{1}{100\;000}\;\sum_{i=1}^{100\;000}\;{\left(\hat{p_{i}}-p\right)}^{2}}$$

To consider the divergence of the true prevalences of the indicators, the normalized root-mean-squared error (NRMSE) was chosen as a measure of accuracy. It is defined as follows:

(4) $$\mathrm{NRMSE}=\frac{\mathrm{RMSE}}{\max\left(\hat{p_{i}}\right)-\;\min\left(\hat{p_{i}}\right)}$$

The R-package SimDesign version 1.11 [7] was used to calculate both RB and NRMSE. Both measures are dimensionless. RB has values between −1 and (1 − *p*)/*p*, with *p* being the population prevalence. Negative values of RB indicate a systematic underestimation of an indicator; likewise, positive values indicate a systematic overestimation, with RB = 0 meaning the absence of bias. NRMSE has values ≥ 0. The larger the value of NRMSE, the greater is the inaccuracy.

## 3. Results

### 3.1. Comparison of the True and the Estimated Prevalence of the Animal Welfare Indicators

**Figure 1:**
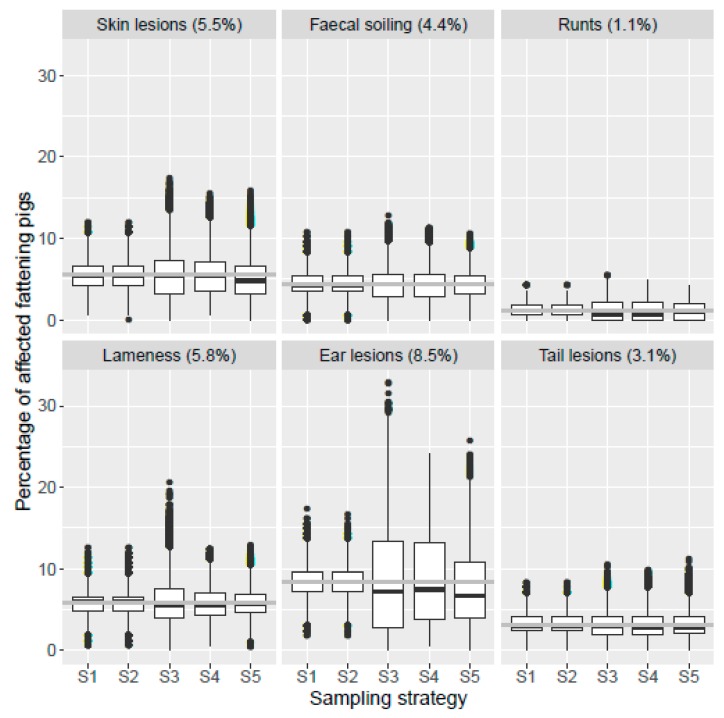
Comparison of the true prevalences of the indicators determined in a full census, i.e., the animal welfare assessment of all fattening pigs in a herd (represented by the gray line; the values are given in brackets), and the estimated prevalences based upon samples of the fattening pigs that were drawn according to five strategies S1–S5 (represented by the box plots; n = 100,000 samples per strategy and indicator, S1 = simple random sampling, S2 = stratified random sampling, S3 = clustered sampling, S4 = stratified clustered sampling, S5 = the KTBL method).

The results of the welfare assessment of all 636 fattening pigs, i.e., the true prevalence of the KTBL indicators, are shown in Figure 1 (represented by the line and indicated behind the name of the indicator). For each indicator, the proportion of affected fattening pigs in the herd is given as a percentage, i.e., the proportion of pigs rated with the highest grade of the indicator (Score 1 for tail lesions, ear lesions, runts, and lameness; Score 2 for skin lesions and faecal soiling). The highest true prevalence occurred with the indicator ear lesions (8.5%). Lower true prevalences were found for the indicators lameness (5.8%), skin lesions (5.5%), faecal soiling (4.4%), tail lesions (3.1%), and runts (1.1%). In addition, Figure 1 shows the estimated prevalence of these indicators, i.e., the proportion of fattening pigs with the highest indicator scores in 100,000 random samples. For each indicator and strategy, box plots represent the scatter of the results of the 100,000 samples. It can be seen that the disparity between the true prevalence of the indicators and the median of the estimated prevalence depends on the strategy. For example, for the indicator ear lesions, the median of the samples collected according to S1 and S2 hits the true prevalence fairly accurately, while the medians of the samples according to S3, S4, and S5 are below the true prevalence. Furthermore, the box plots show the large scatter of the results derived from the 100,000 samples drawn for each indicator and strategy. In some samples, the true prevalence of the indicators has been overestimated by a factor of 2 (indicator ear lesions in S2) to 5 (indicator runts in S3). In other samples, there are no abnormalities which results in an estimated prevalence of 0. This means that the true prevalence is clearly underestimated.

### 3.2. Extent of the Deviation from the True Prevalence of the Animal Welfare Indicators

**Figure 2:**
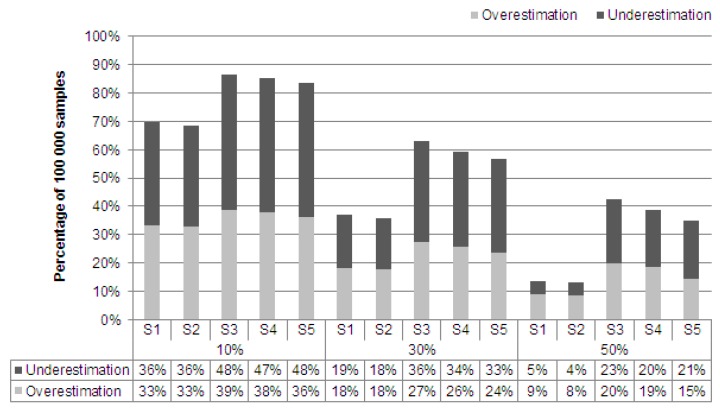
Averaged percentages of the 100,000 samples taken according to the five strategies S1–S5 that over- or underestimated the true prevalences of the six animal welfare indicators for fattening pigs by 10%, 30%, and 50% (S1 = simple random sampling, S2 = stratified random sampling, S3 = clustered sampling, S4 = stratified clustered sampling, S5 = the KTBL method).

The descriptive comparison showed that there were differences between the true and the estimated prevalence of the six indicators considered. The question arises as to the extent of the differences between the true and the estimated prevalences. Figure 2 shows the percentage proportion of the 100,000 samples collected per strategy, averaged over all six indicators, which under- or overestimated the true prevalence by 10%, 30%, and 50%. It is conspicuous that (1) depending on the strategy, between 69% (S2) and 87% (S3) of the random samples deviated by more than 10% from the true prevalence of the indicators. The fractions of the samples that lead to an incorrect assessment of the animal welfare level of the entire herd by more than 30% are between 36% (S2) and 63% (S3). Moreover, between 12% (S2) and 43% (S3) of the 100,000 samples collected deviated from the true prevalence by more than 50%. (2) When looking at the deviation according to an under- or overestimation, it became apparent that the proportion of underestimated samples is almost always larger than the proportion of overestimated samples (S1 and S2 in relation to the proportion of samples deviating from the true prevalence by more than 50% are an exception). The true prevalences of the KTBL indicators therefore tended to be underestimated rather than overestimated by the sampling. (3) A comparison of the five strategies under investigation clearly showed that the samples taken according to S1 or S2 deviated from the true prevalences of the indicators to a much lesser extent than the samples generated according to S3, S4, or S5.

**Figure 3:**
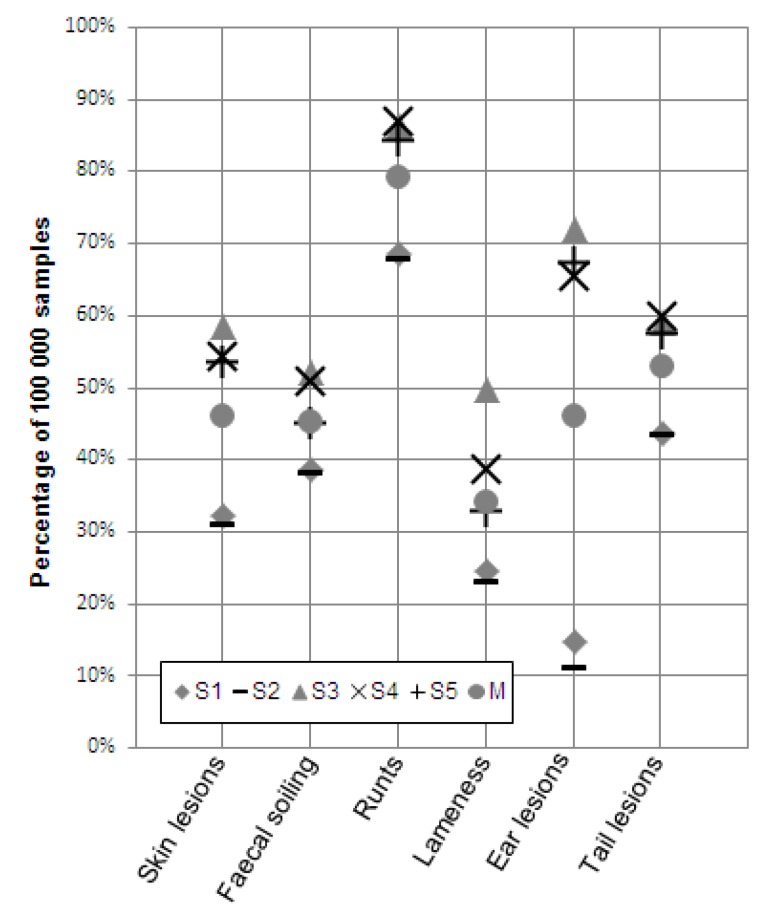
Proportions of the 100,000 samples collected for each strategy (S1–S5) and each welfare indicator for fattening pigs that under- or overestimated the true prevalence by more than 30% (under- and overestimating samples considered in total; M is the average percentage for each indicator over all five strategies, S1 = simple random sampling, S2 = stratified random sampling, S3 = clustered sampling, S4 = stratified clustered sampling, S5 = the KTBL method).

Figure 3 provides detailed information about the differences between the indicators with respect to the degree of deviation from the true prevalences. In this figure, the percentage of the 100,000 random samples that deviated from the true prevalence by more than 30% is shown for the six indicators taken according to the five strategies (the over- and underestimations are considered as a sum). In addition, the average percentage of all samples that deviated by more than 30% over all five strategies are shown for each indicator. This shows that, on average, across all five strategies, some indicators had a larger proportion of misjudged samples than others. The lowest average degree of deviation from the true prevalence over all five strategies occurred with the indicator lameness (34%). The indicator runts, in contrast, had the highest average deviation from the true prevalence over all five strategies (79%). The average proportions of samples that deviated by more than 30% from the true prevalence in the other four considered indicators lay between 45% and 53%.

Within the indicators, the proportion of the samples with more than 30% over- or underestimation of the true prevalence varied according to the sampling strategy. For the indicators faecal soiling, runts, and tail lesions, the range between the strategy with the highest and the lowest proportion of deviated samples lay between 14% and 19%, while the range for the indicators skin lesions and lameness was 27%. With 61 percent points, the indicator ear lesions showed the greatest range, namely between S3, where more than 72% of the samples deviated from the true prevalence by more than 30%, and S2 with 11% over- or underestimated samples. Additionally, Figure 3 shows that for all indicators there is only a slight difference between the strategies S1 and S2 (0–4 percentage points difference in the proportion of samples that deviated from the true prevalence by more than 30%). Within the indicators, the differences between the proportion of samples according to the strategies S3, S4, and S5 that deviated from the true prevalence by more than 30% tended to be slightly larger (for example, 17% for the indicator lameness).

### 3.3. Validity of the Five Random Sampling Strategies

**Figure 4:**
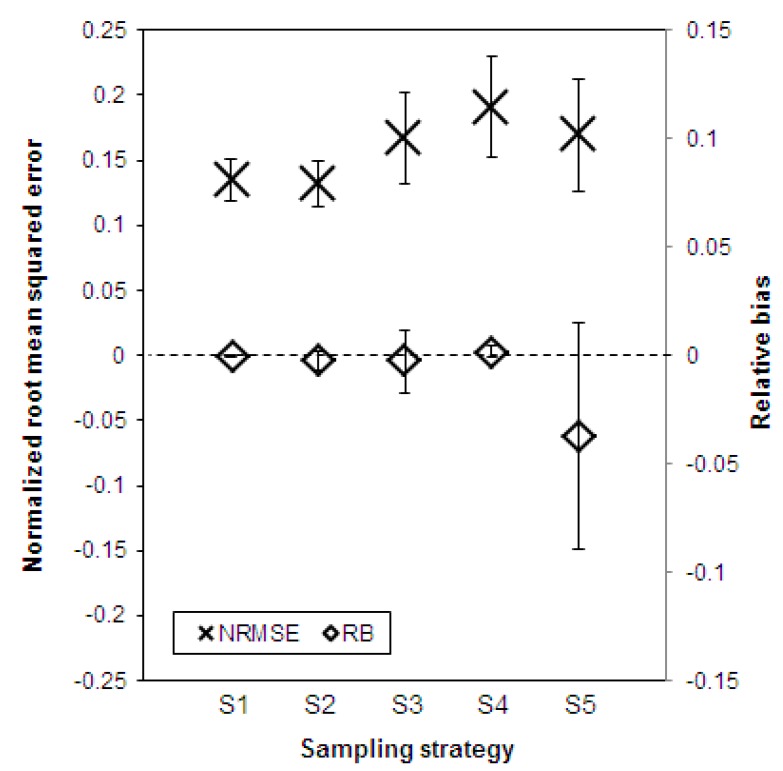
Validity of the 100,000 samples collected according to five strategies (S1–S5) evaluated by the accuracy (NRMSE, normalized root-mean-squared error) and the bias (RB, relative bias) averaged over all six welfare indicators for fattening pigs as well as standard deviation to indicate the differences between the indicators (S1 = simple random sampling, S2 = stratified random sampling, S3 = clustered sampling, S4 = stratified clustered sampling, S5 = the KTBL method).

For the assessment of the validity of the sampling methods, the NRMSE and RB were used to express the accuracy and the bias. Both measures are shown in Figure 4 for all five sampling strategies under investigation and averaged over all six indicators that were considered. The accuracy reflects the degree of scatter of the estimated prevalence of the indicators around the true prevalence. With respect to this, Figure 4 shows that the values obtained with the five sampling strategies differed only slightly. The strategy with the highest degree of accuracy, i.e., the strategy with the lowest degree of scatter across the 100,000 random samples, is S2 (NRMSE = 0.13), while the strategy with the lowest degree of accuracy is S4 (NRMSE = 0.19). The degree of accuracy of the other three strategies lies within this range (for S1, NRMSE = 0.14; for both S3 and S5, NRMSE = 0.17). The standard deviations in Figure 4 indicate the differences within the strategies but between the six indicators considered. It can be seen that the accuracy within strategies S1 and S2 varies between the six indicators slightly less than for strategies S3, S4, and S5.

**Figure 5:**
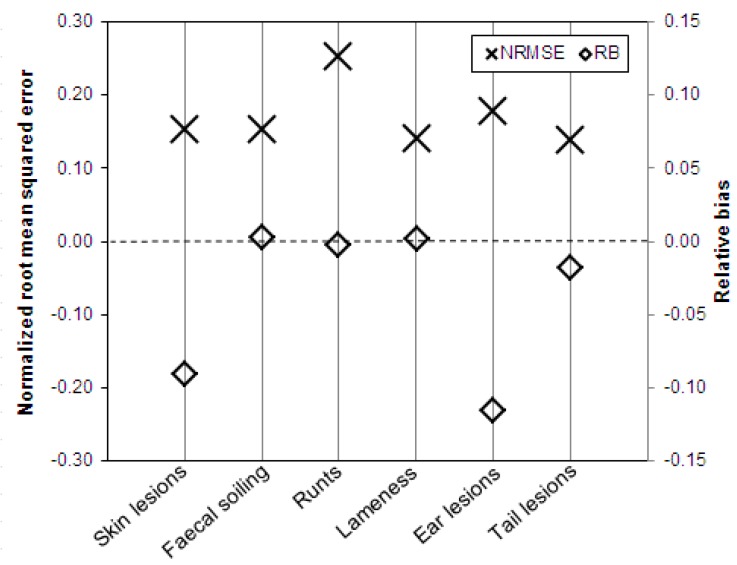
Validity of the 100,000 samples of fattening pigs drawn according to strategy S5 (the KTBL method) and evaluated using six animal welfare indicators assessed by the measures accuracy (NRMSE, normalized root-mean-squared error) and bias (RB, relative bias).

The bias represents systematic deviations between the estimated and the true prevalence of the indicators. In this respect, the following assessment of the five sampling strategies can be made: The samples of fattening pigs collected according to S1 have the least systematic deviation from the true welfare level of the whole herd and lead to a minimal underestimation of the true prevalence of the indicators (RB = −0.0002). The second lowest systematic bias, but with a slightly overestimating tendency, is in the samples selected according to S4 (RB = 0.0017). The strategies S2 and S3 have a similar systematic bias (S2: RB = −0.0025 and S3: RB = −0.0028). S5 is the strategy that has led to samples with the highest degree of bias. Compared to the other sampling strategies, the S5 samples clearly underestimated the true prevalence of the indicators (RB = −0.0370). Particularly obvious is the extremely large standard deviation of the RB in S5, which indicates the variation within the strategy between the six indicators. Therefore, Figure 5 shows the two measures NRMSE and RB for each of the considered animal welfare indicators within strategy S5. While the NRMSE did not vary to any great extent (between NRMSE = 0.14 for lameness and tail lesions and NRMSE = 0.25 for runts), the RB showed a great degree of variation between the indicators. It is noticeable that the results for the two groups of indicators, for which different sampling approaches are proposed according to strategy S5 (see Table 3), differed greatly in their RB levels. The three indicators faecal soiling, runts, and lameness, determined according to strategy S5 and thus in accordance with the procedure recommended by the KTBL guide for all pigs in ten pens chosen randomly, have a very low systematic bias in comparison with the true prevalence after evaluation of the entire herd (indicator faecal soiling: RB = 0.0026, indicator lameness: RB = 0.0021, and indicator runts: RB = −0.0027). The three indicators skin, ear, and tail lesions recorded in accordance with strategy S5 for 15 randomly selected fattening pigs in each of the chosen ten pens have a clearly greater systematic bias with a tendency to underestimate the true prevalence (indicator skin lesions: RB = −0.0905, indicator ear lesions: RB = −0.1156, and indicator tail lesions: RB = −0.0180).

## 4. Discussion

### 4.1. Comparison with the Sample Validity of Other Animal Welfare Indicators

The aim of the study is to determine whether it is permissible, in terms of validity, to assess random samples of fattening pigs in a herd in the animal welfare assessment. The results show that the evaluation of a sample of the pigs can lead to a considerably different result from the animal welfare assessment of all individuals in a herd. Consequently, the following question arises: what are the results of other studies that have examined the sample validity of animal welfare indicators? Although the collection of indicators for samples of animals from herds is also recommended in other animal welfare assessment schemes, this subject is only addressed in a few scientific studies [8,9,10]. In the well-known *Welfare Quality^®^ Animal Welfare Assessment Protocol for Growing Pigs*, for example, it is recommended to assess a total of 150 fattening pigs from ten pens (in order to evaluate the indicators ‘absence of manure on the body’, ‘wounds on the body’, ‘tail biting’, and ‘lameness’) [2]. In contrast, according to the instructions in the *Bristol Welfare Assurance Programme* for the evaluation of the welfare of breeding sows and fattening pigs, 20 individual animals per age group should be assessed using the indicators soiling of the flanks and hindquarters, tail lesions, and lesions of the head, nape, or flank [3]. However, Leeb et al. [3] stated that, especially for the indicator tail lesions, a large sample is necessary to record a trustworthy value for the prevalence and that this indicator should best be assessed at the slaughterhouse. Studies on the automatic monitoring of different lesions in fattening pigs at the slaughterhouse are already available (for example [11]). Mullan et al. [8], who examined the effect of sampling on the assessment of the welfare of fattening pigs on six farms using the indicators dirtiness, body lesions, tail lesions, bursae, lameness, oral behavior, and pigs requiring hospitalization, came to a similar conclusion. Among other things, the result of this study was that indicators with low prevalences in the entire herd cannot be precisely measured, even with large sample sizes.

According to another study, it is sufficient to evaluate half to one third of the fattening pigs in a room in order to determine lesions of the body, tail and ear correctly ([9] cited in [10]). Thus, in the present study, the indicators should have been collected for a total of 212 to 318 fattening pigs from the four rooms (Table 2 shows the number of fattening pigs per room). Instead, the samples consisted of ≤167 fattening pigs depending on the strategy being used. In the course of further studies, therefore, consideration should be given to an extension of the sample size and the recommendation of the KTBL guide for a static sample size of 150 fattening pigs per herd should be critically reconsidered. As an alternative, investigations should be made to determine the specific percentage of the pigs in a herd that need to be assessed to gain valid information about the animals’ welfare as choosing a percentage would reflect the highly variable sizes of pig herds between farms. A large number of studies on how to deal with random samples are available in the field of wildlife research, where the true prevalence and distribution of characteristics in populations are mainly unknown. In this area of research, the statistical method of bootstrapping is used to investigate the effects of random sampling (e.g., [12,13,14]).

### 4.2. Reflections on the Procedure

The present study deals with a previously neglected topic and is to be understood as a first step towards the investigation of the sample validity of the KTBL animal welfare indicators. An especially positive aspect of this study is the large number of 100,000 random samples collected per strategy and indicator. However, it should be noted that, according to their design, the different weight classes of the fattening pigs should have been taken equally into consideration when generating the samples in S2, S4, and S5. For S2, the 167 fattening pigs are randomly selected from the four rooms (each equivalent to a certain weight class), the number of animals in the sample being proportional to the number of animals in the rooms. For S4 and S5, two pens per room were randomly selected, and, for S5, two additional randomly chosen pens from two different rooms were considered. In principle, such an approach provides a simple and feasible method for obtaining a good cross-section of the pigs in a herd for a welfare evaluation. However, on the day when the true prevalences of the six indicators in the Relliehausen Experimental Farm were recorded, a quite divergent number of fattening pigs were housed in the four rooms (Table 2). This circumstance was just taken into account for S2, but not for S4 or S5, so that, disproportionately, a large number of fattening pigs from room 1 and fewer fattening pigs from room 4 were considered in these two strategies. As the pigs in room 4 were on the fifth day of the fattening period, the skin lesions caused by the ranking fights resulting from the regrouping were taken into account when determining the true prevalence but can be slightly underestimated in the estimated prevalence. The same happens for S3. In S3, eight pens are to be selected randomly without regard to the rooms. Since each room contains eight pens and the selection of each pen is equally probable, the samples generated according to S3 theoretically also contain a disproportionately large number of fattening pigs from room 1. As this example shows, it must be generally clear that the requirement to take the different weight classes proportionally into account is not easy to implement and may result in a considerable need for guidelines and tools for implementation.

In general, the question is how to equally consider different weight classes in samples on farms that do not manage the fattening rooms and pens using the all-in/all-out principle, but which are continuously adding new fattening pigs. For such cases, exact guidelines are necessary for the implementation of the sampling and digital tools for the simple execution of those sampling strategies. Another weakness of this study is that the simulation did not take into account the fact that the fattening pigs are clustered into individual rooms and pens, which means that the result of the animal welfare assessment of pigs of the same room or pen may not be independent. This should be considered in further investigations that are necessary to verify the results of this study. In the course of further investigations on this topic, the data from other fattening pig herds with different true prevalences of the animal welfare indicators and different herd sizes should be used. As mentioned above, further studies should focus not only on identifying the best strategy for the sampling of the fattening pigs from a herd, but also on analyzing the sample size needed to measure the prevalences of the indicators accurately to a predefined threshold.

### 4.3. Feasibility of the Five Random Sampling Strategies

The random samples generated by S1 and S2 deviated from the true prevalences of the indicators to a much lesser extent than those generated by S3, S4, or S5. However, it is crucial to emphasize that the samples generated according to S1 and S2 are truly randomly and not just haphazardly chosen samples. The difference between a haphazard selection and a truly random selection is that, in a haphazardly chosen sample, the person undertaking the welfare evaluation enters a room or pen and chooses, supposedly at random, individual fattening pigs or pens. The person’s attention is always influenced. For example, it could be that lame fattening pigs with reduced activity may be disregarded, or pigs with obvious wounds or bleeding lesions may be given special attention. An independent and truly random sample of fattening pigs or pens has to be defined before the assessor enters the barn without the influence of any background information, e.g., about pens with problems. For this purpose, a simple tool such as an Excel file or application software (app) could be developed and made available in addition to the KTBL guide.

The generation of a statistically sound sample of fattening pigs is one topic, but the practical feasibility is another. Firstly, it must also be possible to locate the pigs contained in a pre-defined sample, which is difficult to do without the widespread use of individual animal identification in pig fattening, for example, by eartags. Secondly, the feasibility of S1 and S2 is considered critical because the number of fattening pigs to be selected can be chosen from all available pens. It is not unlikely that the sample includes fattening pigs from all pens on a farm, which means that the assessor needs to visit a large number of pens during the welfare evaluation. Depending on the distances between the individual pens, this increases the time required and thus also the costs of the animal welfare evaluation. According to Courboulay and Foubert [10], the costs of assessing the welfare of fattening pigs depend mainly on the time required to record the individual indicators.

For S3, S4, and S5, individual pens are selected in the sampling process and are taken into account in the animal welfare evaluation. The feasibility of these three strategies is therefore considered to be higher than that of S1 and S2. Strategy S5, which collects samples in accordance with the recommendations of the KTBL guide, makes a distinction and proposes a different approach for two groups of indicators after selecting individual pens (Table 3). The execution of S5 can be considered complicated because of this distinction. Since the validity of the samples generated according to S5 is not higher than that of the samples collected according to S3 or S4, consideration should be given to changing the procedure recommended in the KTBL guide for the sampling of fattening pigs in favor of the simpler strategies S3 or S4. However, this requires that the results of this case study can be confirmed in further investigations using data from farms.

## 5. Conclusions

This study deals with the subject of sample validity in animal welfare evaluation. In many animal welfare assessment schemes, it is proposed to collect indicators for samples of animals from herds, which makes the topic highly relevant. The hypothesis was that despite the evaluation of samples of the fattening pigs, the true animal welfare level of the entire herd can be correctly judged. Based on the results of this study, the hypothesis has to be rejected because the result of the animal welfare assessment of the samples of pigs is partly considerably different from the animal welfare level of the entire herd. Another result is that the five sampling strategies under investigation vary in their feasibility and validity. The results indicate that strategies in which pens are randomly selected and all the pigs in them are assessed could be both more valid and feasible. As this case study only includes data from one farm, this assumption has to be checked in further studies. Data from more farms with differing herd sizes and varying prevalences of the indicators are needed for verification. There is also a need for studies on the valid size of the samples, i.e., what proportion of animals in a herd should be considered in the animal welfare assessment. If the results should be confirmed in further studies, a change of the recommendations in the KTBL proposal *ANIMAL WELFARE INDICATORS: PRACTICAL GUIDE–PIGS* on animal welfare assessment of fattening pigs should be considered.

## Figures and Tables

**Table 1:** Description of the six Kuratorium für Technik und Bauwesen in der Landwirtschaft (KTBL) indicators used in the present study for the welfare assessment based upon samples of fattening pigs from a herd (according to Schrader et al. [1]).

Indicators	Score	Description
Tail lesions	0	No observable wounds/scabs/swellings
	1	Clearly visible wounds/scabs/swellings
Faecal soiling	0	< 10% of the body surface is soiled with faeces
	1	10 to 30% of the body surface is soiled with faeces
	2	> 30% of the body surface is soiled with faeces
Skin lesions	0	< 4 linear lesions with a length ≥ 5 cm and no lesions with a diameter ≥ 2.5 cm
	1	4-15 linear lesions with a length ≥ 5 cm and no lesions with a diameter ≥ 2.5 cm
	2	> 15 linear lesions with a length ≥ 5 cm or at least one lesion with a diameter ≥ 2.5 cm
Ear lesions	0	No lesions or only some scratches on the external ear lobe but no larger wounds or scabs
	1	Clearly visible haemorrhagic wounds and scabs
Lameness	0	No or only a slight degree of lameness (stiff gait, fore- shortened stride, snake-like movements of the spine)
	1	Obvious lameness (slight to severe reduction in weight-bearing) or ‘downer’ pig
Runts	no	Pig is clinically unremarkable
	yes	Pig shows at least two of those four characteristics: much smaller than the other animals in its group, protruding vertebrae, sunken flanks, long bristles

**Table 2:** Number of fattening pigs assessed per room with the average body weights and the average day of fattening.

Room	Number of Fattening Pigs	Average Live Body Weight (kg)	Day of Fattening ^1^
1	88	103.8	84th
2	174	57.1	32nd
3	174	84.9	63rd
4	200	32.5	5th

^1^ number of days since the pigs were moved to the fattening pens.

**Table 3:** Description of the five strategies tested for the collection of the samples of the fattening pigs in a herd.

Strategy	Description
S1	Simple random sampling: 167 pigs chosen randomly from the herd
S2	Stratified random sampling: 167 pigs randomly selected from the herd, weight classes taken into account proportionally

S3	Clustered sampling: random choice of 8 pens, whose pigs are all evaluated
S4	Stratified clustered sampling: random selection of eight pens taking into account the different weight classes; all the pigs in these pens are evaluated

S5	Method according to the KTBL guide for herds > 150 pigs: the random selection of ten pens taking into account the different weight classes; if > 15 pigs per pen: runts, faecal soiling and lameness are evaluated in all pigs in those ten pens; skin lesions, ear lesions and tail lesions assessed on 15 pigs chosen randomly from those ten pens; if < 15 pigs per pen: all six indicators are evaluated in all the pigs in those ten pens

